# The 21st century epidemic: infections as inductors of neuro-degeneration associated with Alzheimer’s Disease

**DOI:** 10.1186/s12979-014-0022-8

**Published:** 2014-12-05

**Authors:** Federico Licastro, Ilaria Carbone, Elena Raschi, Elisa Porcellini

**Affiliations:** Department of Experimental, Diagnostic and Specialty Medicine, School of Medicine, University of Bologna, Bologna, 40100 Italy; Laboratory of Immunopathology and Immunogenetics, Department of Experimental, Diagnostic and Specialty Medicine, School of Medicine, University of Bologna, Via S. Giacomo 14, 40126 Bologna, Italy

**Keywords:** Alzheimer’s disease, Herpes virus latency, Peripheral inflammation, Neuro-inflammation, Inflammatory markers and cognitive decline

## Abstract

Alzheimer’s disease (AD) is a complex disease resulting in neurodegeneration and cognitive impairment. Investigations on environmental factors implicated in AD are scarce and the etiology of the disease remains up to now obscure. The disease’s pathogenesis may be multi-factorial and different etiological factors may converge during aging and induce an activation of brain microglia and macrophages. This microglia priming will result in chronic neuro-inflammation under chronic antigen activation. Infective agents may prime and drive iper-activation of microglia and be partially responsible of the induction of brain inflammation and decline of cognitive performances. Age-associated immune dis-functions induced by chronic sub-clinical infections appear to substantially contribute to the appearance of neuro-inflammation in the elderly. Individual predisposition to less efficient immune responses is another relevant factor contributing to impaired regulation of inflammatory responses and accelerated cognitive decline.

Life-long virus infection may play a pivotal role in activating peripheral and central inflammatory responses and in turn contributing to increased cognitive impairment in preclinical and clinical AD.

## Introduction

### Alzheimer dementia type and infections

Alzheimer’s disease (AD) is a progressive neurodegenerative disorder and the most common cause of dementia. According to the World Health Organization, nearly 35.6 millions of people worldwide currently may suffer from dementia. The disease affects people in all countries with more than half patients living in low- and middle-income countries and by 2050, this figure is likely to rise to more than 70% [1].

Because of the urgency for effective preventive and therapeutic measures, extensive research has focused on pathogenetic mechanisms of AD, however, up to now, no therapy has been found.

Some neuro-pathological alterations such as amyloid deposition and neurofibrillary tangles (NFTs) are almost always found in the brain after the autopsy examination of patients who suffered dementia. Therefore, these alterations have been suggested to be causative of the disease [2,3]. However, these pathological alterations are also present in the brain of elderly who died without the clinical presentation of AD [4] and the notion that amyloid deposition and other proteinaceous alterations might be causative of AD is made uncertain by these observations.

Amyloid beta peptide (Abeta) is the major component of amyloid deposits in AD brains [2] and derives from the processing of a highly conserved membrane protein named amyloid precursor protein (APP) [5]. The physiological function of APP and its biological role remain unclear [6]. However, few years ago a role as anti-microbial defensive factor for A-beta peptide has been suggested [7]. A recent investigation confirmed that the A-beta peptides showed a relevant anti-virus activity *in vitro* and suggested that these peptides may have a defensive role against the influenza virus [8].

## Review

### Herpes family and dementia

In our previous publications [9,10] we discussed genetic data from four genome wide association (GWA) studies on AD [11–14]. From these investigations a set of single-nucleotide polymorphisms (SNPs) associated with AD emerged and we suggested that the concomitant presence of these SNPs might result in a genetic signature predisposing to AD, via complex and diverse mechanisms, each contributing to an increase of individual susceptibility to herpes virus infection [9,10].

A viral etiology, especially involving herpes virus in AD, has been already proposed and most investigations have shown an association of herpes simplex virus type 1 (HSV-1) with AD [15–19].

HSV-1 is a ubiquitous virus that affects more than 80% of people over 65 worldwide. It is a neurotropic double-stranded DNA virus that primarily infects epithelial cells of oral and nasal mucosa. Here virus undergoes lytic replication; the newly produced viral particles may enter sensory neurons and, by axonal transport, reach the trigeminal ganglion where usually establishes a latent infection. The virus undergoes periodic reactivation cycles in which the newly formed viral particles are transported back to the site of primary infection through the sensory neurons, causing the well-known clinical lesions (i.e., cold sores and blisters). However, the bipolar trigeminal ganglion neurons also project to the trigeminal nuclei located in the brainstem. From here, neurons project to the thalamus to finally reach the sensory cortex. This is the path through which the reactivated virus may reach the central nervous system (CNS), where it may cause acute neurological disorders like encephalitis [herpes simplex encephalitis (HSE)] or a mild, clinically asymptomatic, infection, or establish life-long latent infection [20].

Recent reports showed a significant association of HSV-1 infection with AD risk in a longitudinal nested study from Sweden [21]. A reactivation of HSV-1 infection assessed by increased serum levels of specific anti-HSV-1 antibodies was found associated with an increased AD risk in a longitudinal study on 3432 elderly [21]. Another study from Italy reported that elevated serum HSV-1 antibody titers correlated with cortical grey matter volume as assessed by MRI [22].

It is interesting to note that others herpes viruses share the ability to become latent in the infected host and eventually latently infect neurons.

On the other hand, investigations focused on different viruses of the herpes family, such as human cytomegalovirus (CMV), Epstein-Barr virus (EBV) or human herpes virus 6 (HHV-6) in AD are limited.

CMV is ubiquitously distributed in human population and the most frequent brain infection in immune compromised patients or in infants with congenital virus transmission [23]. Postnatal acute peripheral CMV infection is usually asymptomatic, but once established, the virus remains latent in blood monocytes [24].

CMV has also been associated with other chronic diseases of aging, including cardiovascular disease, cognitive decline and cancer. The specific mechanisms responsible for these associations have not been fully understood, but they are likely to have an immune and inflammatory component [25].

The sero-conversion to positive CMV may vary over the years, ranging between 0.5 to 1.5% per year. It has been suggested that CMV is responsible for the age-associated immune changes in the elderly which lead to a reduction in the number of naïve T cells [26,27].

An increased rate of cognitive decline over a four year period in subjects with elevated CMV antibody levels has also been reported [28]. Previous work upon brain frontal and temporal cortex samples found that both AD patients and elderly healthy subjects were positive for CMV with no statistically significant difference between the two groups [29]. CMV was found in the brain of a greater proportion of patients with vascular dementia than normal elderly; these findings suggested a role for this virus in the disease [30].

Our recent work showed that increased CMV antibody levels were present in the elderly who developed clinical AD during a five years follow up [31].

Findings from another investigation reported that CMV infection doubled the risk of developing AD in a longitudinal follow up of 849 participants from USA [32].

EBV infects more than 95% of human beings within the first years of life. The virus causes acute infectious mononucleosis in a minority of immune competent subjects, while the majority develops a lifelong asymptomatic infection and the virus remains latent in B-lymphocytes. EBV is also involved in the development of several diseases such as Burkitt lymphoma, Hodgkin lymphoma and nasopharyngeal carcinoma [33].

Moreover, EBV seems to be involved in the pathogenesis of various neurological diseases, such as encephalitis, neuritis, myelitis, cerebellitis, acute disseminated encephalomyelitis, or central nervous system (CNS) lymphoma in patients with the immunodeficiency virus (HIV) infection [34] and multiple sclerosis [35].

Recently our findings showed an association of peripheral blood positivity for EBV genome and AD [31]. Moreover, elevated levels of EBV specific antibodies were associated with an increased AD risk [31].

HHV-6 is a neurotropic virus and has been associated with multiple neurological diseases including seizures, encephalitis, mesial temporal lobe epilepsy and multiple sclerosis [36].

HHV-6 has been found in a higher proportion of AD brains than age-matched control (CTR) brains [29]. However, these findings were not confirmed by another investigation [37] that reported a higher value of HHV-6 level in CTR brains.

Our findings showed an elevated positivity in brains and peripheral blood for HHV-6 genome in AD [31]. Increased sero-positivity was also associated with clinical diagnosis of AD [31].

The sero-positivity to CMV, EBV or HHV-6 is very high worldwide and viruses of the herpes family are largely and commonly present in the elderly.

It is of interest to note that the immune response of the host to infections undergoes age-dependent changes following a process called immune senescence. Immune senescence may lead to an increased susceptibility of older adults to develop, not only infectious disease, but also Alzheimer’s disease, osteoporosis, cancer and autoimmunity [38].

### The impact of persistent virus infections upon the impairment of immune responses in the elderly

The aging of the immune system is a continuous and dynamic process and it might be secondary to mechanisms activated by the response to the pathogen individual internal milieu [39].

No all immune responses show the same rate of aging or senescence. In fact, innate immunity seems to be preserved along the years, while adaptive immune responses progressively decline with age [40].

Recent investigations focused on immune senescence suggested that the progressive decline of immune defense efficiency might be an adaptation mechanism to the microorganism exposure experienced by the aging organism over the life time [41].

A pivotal question, therefore, is: what is the cause of the progressive senescence of the adaptive immune responses in the elderly?

Longitudinal investigations showed that T cell phenotypes and functions progressively change with advancing age and these populations in the peripheral blood of the elderly consist of super specialized CD4 positive and CD8 positive T lymphocyte populations [42,43]. These T cell populations appeared to be immunologically exhausted according to some Authors [43].

It is interesting to note that the common presence of CMV sero-positivity in the elderly is associated to an age related increase of specialized CD8T cells specific for CMV antigens [44]. Moreover, other chronic virus infections also contribute to shape the immune phenotype during aging and their collective immune pressure changes the representation of peripheral T cell populations in the elderly [41].

Naïve T cells are positive for the following surface markers: CCR7, CD45RA, CD27 and CD28. Central memory T cells (CM) are positive for CCR7, CD27 and CD28 markers. Memory T effector cells (EM) are lightly positive for CD27 and CD28. Finally, the terminal differentiated memory T cells (TEMRA) are positive for CD45RA and KLRG-1 surface markers [41].

A summary of the immune impact of virus infection on human circulating T cells representation is reported in Table 1 [41].

**Table 1:** **Effects of persistent virus infections upon the different T cell populations in the peripheral blood of young and old subjects [modified from Fülöp et al** [41]

	**CMV**	**HBV**	**EBV**	**VZV**	**HSV-1**	**HHV-6**
Expansion	+++	+	+	-	-	?
Viral load	+/−	+/−	+/−	+/−	+/−	+/−
Reactivation	?	?	?	+/−	+/−	-
Immune phenotype	TEMRA	EM	EM	CM/EM	EM	TREG
Immunological aging	++++	+	++	-	-	-
Clinical impact in young	Moderate	Mild	Moderate	Mild	Mild	Moderate/Severe
Clinical impact in elderly	Moderate	Mild	Moderate	Severe	Mild	?

CM = central memory cells; EM = effector memory cells; TEMRA = terminally differentiated memory cells re-expressing CD45RA.

? = data not available.

EBV, varicella zoster virus and HSV-1 have a severe impact on immune system and contribute to reshape the immune phenotype in the old person by inducing a persistent antigenic stimulation [45].

For instance, viruses of the herpes family infect the majority of human population since childhood and by frequent cycle of reactivation and latency constantly challenge the immune response and drive the accumulation of memory T cells. Therefore, the continuous antigen stimulation induced by chronic infectious microorganisms activates a peripheral chronic inflammatory response that progressively induces the loss of naïve and inducible CD4 and CD8 positive T cells and the accumulation of memory T cell populations.

**Figure 1:**
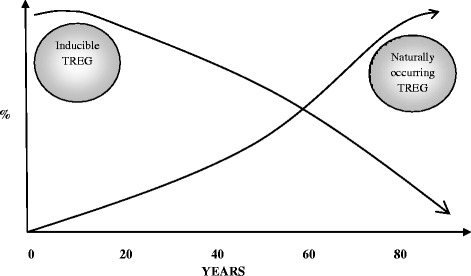
**The change in the percentage of regulatory T cells (Tregs) with the age [modified from Fülöp et al.** **[** 41 **].**

The change in the percentage and the absolute number of regulatory T cells (Tregs) plays a peculiar relevance in the age dependent re-shaping in the human immune phenotype. In fact, a progressive loss of inducible Tregs and an increment of naturally occurring Tregs characterized T cell population change in the elderly, as shown in Figure 1 [41].

We can conclude that during aging a constant antigen pressure is partially responsible for the age associated immune decline.

**Figure 2:**
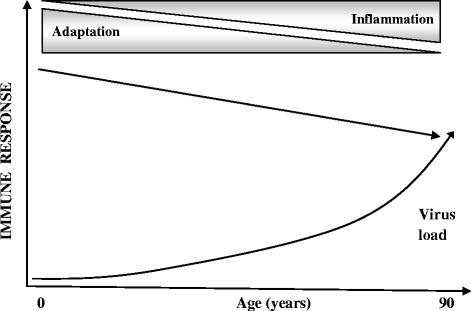
**The decline of the immune diversity and plasticity with advancing age in relation to a decrement of immune reserve induced by antigen load of chronic infections [modified from Fülöp et al.** **[** 41 **].**

In fact, with advancing age the adaptive immune response, the immune diversity and the plasticity of immune responses decline because of the immune reserve decrease induced by antigen load of chronic infections. This process is represented in Figure 2 [41].

Chronic infections represent important environmental factors able to induce a re-shaping of the immune system by antigen load during aging. Chronic sub-clinical viral infections such as those caused by herpes viruses with their characteristic cycles of latency and reactivation may play a relevant role. These viruses indeed infect a large proportion of human population and the immune system is not able to completely eradicate the viruses.

Moreover, as is the case of CMV, the adaptive immune responses pay a high price to maintain the virus in the latent form, since the immune resources involved in anti-virus defensive mechanisms are elevated. In fact, in the elderly as many as 50% of cytotoxic CD8 positive T cells and 30% of the helper CD4 T cells can be positive for this virus antigens. Naïve and memory T cells with different antigen specificity proportionally and concomitantly decrease.

Decreased protection after vaccination, increased risk of cardiovascular diseases and type 2 diabetes are the clinical consequences of the immune system re-shaping in the elderly. All these conditions are associated with increased age-associated peripheral inflammation.

### Classical and alternatively activated microglia in AD

It has been known for some decades that tissue macrophages may be differently activated. These two stages of metabolic activation have been defined M1 and M2 and they can be identified by up-regulation of membrane markers after stimulation by different factors.

M1 macrophages show a pro inflammatory function, whilst M2 have anti inflammatory activity.

IFN-gamma produced by T helper-1 lymphocytes induces the M1 activation state and these macrophages produce high levels of TNF-alpha and iNOS [46].

M2 macrophages, also called alternatively activated, do not release NO, are not cytotoxic, and are activated by T helper- 2 derived interleukins, such as IL-4, IL-5 and IL-13. M2 are considered regulatory macrophages, since they inhibit the release of several cytokines from other cells of the immune system [47].

As far as AD neurodegeneration is concerned, there is a consensus suggesting that the inflammatory milieu associated with the neuro-inflammation inhibits the microglia phagocytosis [48]. Moreover, in the PS1M146L/APP751SL mouse model of AD an age dependent switch of brain microglia from the alternative to the classical phenotype was observed [49].

However, other investigations on AD animal models reported that brain microglia over-expressed markers of the alternatively activated phenotype [50,48].

Under normal conditions, few activated T cells gain entry to the brain and are involved in immune surveillance. However, infiltration of a significant number of T cells occurs in brain disease or after brain injury. The consequences of T cell infiltration may play a neurodestructive or neuroprotective role in different disease animal models [48]. A recent paper reviewed this topic concluding that brain infiltrating T cells regulate microglia activation by releasing IFN-gamma and, therefore, driving neuro-degenerative processes associated with AD [48].

Microglia activation in pre-clinical and clinical AD by neuro-imaging techniques has been reported [51,52].

A defective resolution of inflammatory state has been recently found in the brain of patients with AD and this impairment correlated with cognitive function [53]. Moreover, elevated levels of CNS inflammation and CSF inflammatory markers have been also reported in preclinical stages of AD [54].

In conclusion, brain microglia from AD patients is activated and release several cytokines that induce neuro-inflammation.

### Peripheral inflammation and neurodegeneration in AD

As above discussed, inflammatory responses are present in the brain of patients with AD and activated microglia cells are a pathological marker of the disease [55].

However, it is important to note that several observations have also shown that increased peripheral inflammatory responses are detectable in patients with AD [56]. For instance, increased levels of certain cytokines and acute phase proteins are well detectable in the blood of AD [57]. Therefore, peripheral inflammatory state is higher in AD patients than in cognitively healthy elderly [58,56].

It is likely that, as the neurodegenerative processes progress in the brain, a concomitant increased peripheral dis-regulation of immune responses increases.

This notion is supported by several observations. For instance, a recent report from a study originally designed to investigate of osteoporotic fractures showed that a significant change in peripheral inflammatory markers was present in the oldest-old women and correlated with cognitive decline [59].

A recent overview of the topic concluded that published data were conflicting; however, some cytokines showed a steady increase during progression from mild cognitive decline (MCI) to AD [60].

An association between late life depression, MCI and AD is well documented and an interesting paper suggested that peripheral inflammation might be the missing link in these different conditions [61].

Increased serum levels of inflammatory factors have been reported also in MCI with different genetic background from China [62].

A recent report by applying sophisticated statistical analysis to disclose the relationship between immunological and oxidative stress markers in AD and MCI, showed that a global immune deficit in MCI and AD was detectable [63]. In fact, both adaptive and innate immunity were peripherally defective. A widespread immune deficit, as suggested in the study, is conceivable to be a concomitant factor in disease progression both for inadequate control of local inflammation and for an insufficient supply in repairing factors [63].

We can conclude that peripheral inflammation is indeed present in early stage of AD and is higher than that observed during non pathological aging [60]. Moreover, an altered inflammatory regulation is also present in MCI and correlate with the progression to AD [60].

### The missing link between central neuro-inflammation and peripheral inflammatory state: infectious factors

It has been shown from the Rush Alzheimer’s Disease Center Religious Order that CMV serum levels were associated with NFT in the autopsy brains [64]. It is of interest that the percentages of senescent CD4 and CD8 T cells were higher in CMV sero-positive than in sero-negative subjects and marginally associated with AD diagnosis. Moreover, Lurain and co-workers reported that the infection of human fibroblasts by CMV induced the expression of amyloid beta peptides [64].

Therefore, a more stringent link may bind peripheral and central inflammatory responses in AD.

This link may consist of chronic infections by microorganisms, such as viruses, that are able to constantly impair immune responses.

### Bacterial infection, cognitive decline and dementia

It is important to keep in mind that several pathogens may show the potential ability to dis-regulate the immune responses.

In fact, virus infections are not the unique challenge for the aging immune system. Persistent low level bacterial infections also play a role in inducing chronic inflammation in the elderly. Specifically, oral microbiome and oral infections have been recently reviewed as potential causes of blood brain barrier (BBB) disruption and brain inflammation; these pathogens may also infect the brain via trigeminal and/or olfactory nerves [65].

Chronic inflammation in periodontal disease, for example, has been suggested as a potential risk factor in Alzheimer’s disease [66]. Periodontal disease is a peripheral, chronic infection, which elicits a systemic inflammatory response [67]. The chronic trickling of Gram negative, anaerobic periodontal bacteria into the systemic bloodstream result in elevated levels of various inflammatory mediators in the serum of periodontitis patients. Some inflammatory mediators associated with periodontal disease, e.g. C Reactive Protein (CRP), Interleukin 6 (IL 6), Interleukin 1(IL 1β), and TNF-α have been suggested to increase the risk of cognitive decline and/or Alzheimer’s disease [66].

In the study of Sparks Stein P et al., both the AD and MCI subjects demonstrated significant elevations in antibody to *P. intermedia* and *F. nucleatum* at baseline, prior to diagnosis of the neurological changes. Additionally, the AD subjects expressed significantly elevated antibody to *T. denticola*, and *P. gingivalis* at baseline. Interestingly, the control group also showed antibody levels higher than healthy values for four of the seven bacteria (*A. actinomycetemcomitans*, *C. rectus*, *T. forsythia* and *P gingivalis*) with three of the four at levels consistent with chronic periodontal disease. Regardless, the levels of antibodies in the control group were significantly lower than the levels of those who converted to AD at baseline for five of the seven bacteria studied [66]. Whether oral bacteria themselves or endotoxins (e.g., LPS) released by them gain access to the brain, the net result is likely to be microglial activation. Microglial activation is a well-recognized feature of AD and results in the increased production of proinflammatory cytokines such as TNF and IL1β. This could explain why levels of, for example, TNF in the cerebrospinal fluid of AD patients reach such high levels, 25-fold that of controls. Prolonged exposure to high concentrations of TNF weakens the protective BBB making it more permeable to ingress of bacteria or endotoxins [65].

Chronic low level inflammation induced by sub clinical infections may therefore play a pivotal role in directly or indirectly activate brain immune responses and neuro-inflammation.

### Host genetic makeup, immune responses and dementia

However, it is also relevant how the host responds to these microorganisms. In fact, the individual genetic background plays a pivotal role in the maintenance of the chronic inflammation both in the brain and in the peripheral tissues.

In this context, as already mentioned, GWA studies in AD showed that several immune factors were associated with increased risk of the disease. However, each single immune gene showed a low odd ratio (OR < 1.7) of association with AD.

The only exception was the allele 4 of the APOE gene that was confirmed to have a high OR with AD. APOE gene also appears to be involved in chronic infections and it is important to note that the APOE gene is a well known susceptibility factor for several virus infections [68–70]. Besides, ApoE4 compromises the integrity of the BBB by activating the cyclophilin A matrix metalloproteinase MMP-9 pathway [71]. This is particularly important if the penetration of bacteria or LPS into the brain is involved in the initiation or progression of AD [65].

The weak association of immune genes with AD can be simply explained, as no immune factor is the cause of the disease. Nevertheless, the concomitant presence of several genetic factors in the same individual might show a more sound association and individual infection susceptibility may be affected by the concomitant presence of alleles resulting in decreased immune efficiency [9,10].

As infections appear to play a role, the link between a given pathogen and the host susceptibility to its infectivity might be one missing link in the pathogenesis of cognitive decline progression to clinical AD.

Our recent findings showed that polymorphisms in genes regulating antiviral responses are differently distributed in AD and influence a differential positivity to EBV and HHV-6 genomes in the elderly (data submitted). Moreover, risk alleles were increased in elderly progressing to AD (data submitted). These observations reinforce the notion that individual genetic background plays a role in the progression of cognitive impairment by influencing the efficiency of immune responses to persistent parasites.

Different immune genetic makeup will weaken the defensive mechanisms against few pathogens. This immunological weakness, however, will show relevant effects over the life span time by interfering with the defensive mechanisms against other pathogens able to directly infect the brain or against microorganisms able to produce toxins which, in turn, impair the BBB and/or kill neurons. All these mechanisms may have deleterious effects on cognitive performances during aging.

## Conclusions

AD is a multi-factorial diseases, which shows different etiological and pathogenetic factors. Several different pathogens, both viruses and bacteria, may play a role in triggering ill controlled inflammatory responses and directly or indirectly activate neuro-inflammation. An important role in brain defenses against microorganisms is played by APP and its peptides. However, in subjects who will develop AD this protective mechanism appear to be unsuccessful. Besides, the pathogen induced reshaping of adaptive immune responses has deep consequences in the altered regulation of both peripheral and central immune defensive mechanisms.

Individual susceptibility to different pathogens under the control of personal genetic background plays a secondary but not marginal role in the unsuccessful regulation of defensive immune responses and poorly controlled inflammation. Therefore, the activation of persistent peripheral inflammation has detrimental effect upon the brain in genetically susceptible individuals.

If this view of the disease will be shared by an increasing number of scientists and experimentally and clinically verified, several new therapeutic interventions may open for AD patients. Successful treatment of chronic infections is a challenging but mandatory goal to improve the quality of life in the elderly.
